# Supplementary material for: Manure in combination with optimal topdressing with nitrogen fertiliser improved growth, grain yields and the efficiencies of water and nitrogen use in winter wheat in the Xinjiang Oasis drylands
Source: PeerJ. 2025 Jun 13;13:e19543. doi: 10.7717/peerj.19543 (PMC12169170; doi:10.7717/peerj.19543)
Supplement: Supplemental Information 2 [file peerj-13-19543-s002.docx]

**Supplementary Information (SI)**

**Manure fertilizer addition and optimal topdressing nitrogen input improved growth, grain yield and water-nitrogen use efficiencies of winter wheat in the drylands of the Xinjiang Oasis**

Yanfei Fang^1^, Jianghua Tang^1^, Shanqing Zhang^2^, Na Zhang^3^, Xiaoying Luo^1^, Dongping Hu^1^ and Wenxiu Xu^1*^

*^1^ College of Agronomy, Xinjiang Agricultural University, Urumqi, Xinjiang, China*

*^2^ Xinjiang Uygur Autonomous Region Agro-meteorological Observatory/Information Centre of Xinjiang Xingnong-Net, Urumqi, Xinjiang, China*

*^3^ Institute of Economic Crops, Xinjiang Academy of Agricultural Sciences, Urumqi, Xinjiang, China*

* Corresponding author (Tel: +86-13199847582; Email: [xjxuwenxiu@163.com)](mailto:xjxuwenxiu@163.com))

**Supplementary Table S1.** Significance analysis (*F value*) of the interactive effects of manure fertiliser addition, topdressing nitrogen rates, and year on LAI, AGB, *Pn*, *Tr,* NUA and NUE over 2 years.

| **Parameter** | **LAI** | **AGB** | ***Pn*** | ***Tr*** | **NUA** | **NUE** |
| --- | --- | --- | --- | --- | --- | --- |
| Manure (M) | 242.92** | 106.62** | 0.25ns | 5.58* | 385.81** | 49.40** |
| N rate (N) | 101.24** | 54.86** | 14.86** | 3.47* | 200.92** | 171.03** |
| Year (Y) | 1475.17** | 442.73** | 340.87** | 93.95** | 1662.90** | 103.46** |
| M×N | 6.53** | 1.83ns | 2.79ns | 2.71ns | 2.37ns | 15.18** |
| M×N×Y | 7.36** | 1.28ns | 0.05ns | 0.98ns | 0.40ns | 1.39ns |

**Note:** LAI, leaf area index; AGB, aboveground biomass; *Pn*, net photosynthetic rate; *Tr*, transpiration rate; NUA, crop N uptake; NUE, nitrogen use efficiency; * significant at the 0.05 probability level; ** significant at the 0.01 probability level; ns No significant.

**Supplementary Table S2.** Comprehensive principal component scores for different treatments and rankings.

| 2021-2022 |  |  |  | 2022-2023 |  |  |
| --- | --- | --- | --- | --- | --- | --- |
| Treatment | Score | Rank |  | Treatment | Score | Rank |
| M0N0 | -1.33 | 6 |  | M0N0 | -1.08 | 6 |
| M0N150 | 0.3 | 3 |  | M0N150 | 0.03 | 4 |
| M0N300 | 0.16 | 4 |  | M0N300 | -0.25 | 5 |
| M1N0 | -0.58 | 5 |  | M1N0 | 0.14 | 3 |
| M1N150 | 0.92 | 1 |  | M1N150 | 0.77 | 1 |
| M1N300 | 0.54 | 2 |  | M1N300 | 0.40 | 2 |

**Supplementary Table S3.** The averages of the main effects of manure and topdressing nitrogen fertilizers on LAI, aboveground biomass, *Pn* and *Tr*.

| Years | Manure (kg ha^-1^) | Nitrogen fertilizer  (kg ha^-1^) | LAI | ABG  (kg ha^-1^) | *Pn*  (µmol·m^−2^·s^−1^) | *Tr*  (mmol·m^−2^·s^−1^) |
| --- | --- | --- | --- | --- | --- | --- |
| 2021-2022 | M0 | / | 0.64 | 5082.47 | 13.22 | 3.92 |
|  | M1 | / | 0.81 | 6366.12 | 12.32 | 3.79 |
|  | / | 0 | 0.55 | 4296.70 | 12.40 | 3.82 |
|  | / | 150 | 0.82 | 6454.88 | 13.74 | 4.06 |
|  | / | 300 | 0.81 | 6421.31 | 12.19 | 3.69 |
| 2022-2023 | M0 | / | 1.33 | 8155.48 | 18.36 | 4.90 |
|  | M1 | / | 1.88 | 10320.33 | 19.54 | 5.74 |
|  | / | 0 | 1.33 | 8285.67 | 17.25 | 5.24 |
|  | / | 150 | 1.81 | 10281.53 | 20.38 | 5.65 |
|  | / | 300 | 1.69 | 9146.52 | 19.30 | 5.07 |

**Note:** LAI, leaf area index; AGB, aboveground biomass; *Pn*, net photosynthetic rate; *Tr*, transpiration rate.

**Supplementary Table S3.** The averages of the main effects of manure and topdressing nitrogen fertilizers on grain yield and components.

| Years | Manure (kg ha^-1^) | Nitrogen fertilizer  (kg ha^-1^) | Spike numbers  (×10^4^ ha^−1^) | Grain number (spike^−1^) | 1000-grain weight (g) | Grain yield  (kg ha^−1^) |
| --- | --- | --- | --- | --- | --- | --- |
| 2021-2022 | M0 | / | 180.00 | 37.01 | 29.21 | 1973.97 |
|  | M1 | / | 209.78 | 38.94 | 29.43 | 2302.91 |
|  | / | 0 | 160.22 | 39.77 | 30.83 | 1865.99 |
|  | / | 150 | 218.45 | 36.64 | 28.44 | 2368.24 |
|  | / | 300 | 206.00 | 37.52 | 28.69 | 2181.09 |
| 2022-2023 | M0 | / | 384.95 | 33.44 | 25.25 | 2788.96 |
|  | M1 | / | 457.84 | 34.71 | 22.99 | 3045.36 |
|  | / | 0 | 396.80 | 31.99 | 24.99 | 2784.89 |
|  | / | 150 | 441.07 | 35.90 | 23.10 | 3031.62 |
|  | / | 300 | 426.31 | 34.33 | 24.27 | 2934.97 |

**Supplementary Table S3.** The averages of the main effects of manure and topdressing nitrogen fertilizers on NUA, NUE, ET_c_ and WUE.

| Years | Manure (kg ha^-1^) | Nitrogen fertilizer  (kg ha^-1^) | NUA (kg ha^−1^) | NUE (%) | ET_c_ (mm) | WUE  (kg ha^−1^ mm^−1^) |
| --- | --- | --- | --- | --- | --- | --- |
| 2021-2022 | M0 | / | 61.40 | 19.98 | 29.21 | 1973.97 |
|  | M1 | / | 76.81 | 27.00 | 29.43 | 2302.91 |
|  | / | 0 | 53.75 | / | 30.83 | 1865.99 |
|  | / | 150 | 72.49 | 27.17 | 28.44 | 2368.24 |
|  | / | 300 | 81.09 | 19.81 | 28.69 | 2181.09 |
| 2022-2023 | M0 | / | 104.40 | 29.89 | 25.25 | 2788.96 |
|  | M1 | / | 140.26 | 35.81 | 22.99 | 3045.36 |
|  | / | 0 | 101.58 | / | 24.99 | 2784.89 |
|  | / | 150 | 130.02 | 41.21 | 23.10 | 3031.62 |
|  | / | 300 | 135.38 | 24.49 | 24.27 | 2934.97 |

**Note:** NUA, crop N uptake; NUE, nitrogen use efficiency; ET_c_, the actual evapotranspiration; WUE, water use efficiency.
